# Diagnostic accuracy of the Daye diagnostic tampon compared to clinician-collected and self-collected vaginal swabs for detecting HPV: a comparative study

**DOI:** 10.1128/jcm.01852-24

**Published:** 2025-04-11

**Authors:** Valentina Milanova, Michelle Gomes, Kalina Mihaylova, John Luke Twelves, Jan Multmeier, Hana McMahon, Hannah McCulloch, Kate Cuschieri

**Affiliations:** 1 Daye, London, United Kingdom; 2 Lindus Health, London, United Kingdom; 3 TotaEffects GmH, Oranienburg, Germany; 4 Center for Reproductive Health, University of Edinburgh 3124, Edinburgh, Scotland, United Kingdom; 5 Independent consultant, London, United Kingdom; Mayo Clinic Minnesota, Rochester, Minnesota, USA

**Keywords:** human papillomavirus, HPV, self-sampling for human papillomavirus, self-sampling for HPV, self-testing for HPV, HPV home testing, cervical cancer screening, diagnostic tampon

## Abstract

Cervical cancer screening is vital for achieving global elimination of this preventable disease. Vaginal self-sampling (VSS) for human papillomavirus (HPV) has the potential to increase screening uptake, particularly among individuals who may be underserved by clinician collection. Expanding self-sampling options with accurate, acceptable collection devices is essential. The Daye diagnostic tampon (DDT) offers an innovative approach, utilizing a tampon for HPV-based detection. This study assessed the diagnostic accuracy of DDT in detecting high-risk HPV infections, using vaginal clinician-collected swabs (CCS) as the reference standard. In this UK-based study, 260 participants provided CCS and VSS (with Copan FLOQSwabs) and DDT samples for HPV testing. Samples were analyzed using the Aptima HPV assay, which detects 14 high-risk HPV types. The sensitivity, specificity, positive predictive value, and negative predictive value of the DDT were evaluated against the CCS. Invalidity rates—HPV-negative results with negative internal controls—were compared across sampling methods. The DDT showed a sensitivity of 82.9% [95% confidence interval (CI): 72.4%–89.9%], specificity of 91.6% (CI: 86.4%–94.9%), and overall accuracy of 89.0% (CI: 84.4%–92.4%) relative to CCS. McNemar’s test showed no significant difference between CCS and DDT results (*P* = 0.845). Valid result rates were highest for DDT (99.2%), followed by VSS (95.4%) and CCS (90.8%). The DDT demonstrates comparable accuracy to CCS for detecting high-risk HPV. This novel device shows promise as a self-sampling method. Furthermore, complementary research should focus on assessing DDT’s clinical performance in detecting HPV associated with cervical disease endpoints.

**IMPORTANCE:**

Cervical cancer remains a leading preventable cause of cancer death globally, with persistent disparities in screening access. Self-sampling for HPV has emerged as a critical tool to improve screening uptake, particularly among underserved populations, yet device acceptability and diagnostic reliability remain barriers to equitable implementation. This study demonstrates that the Daye diagnostic tampon (DDT), a novel, tampon-based self-sampling method, achieves diagnostic accuracy comparable to clinician-collected swabs (sensitivity 82.9% and specificity 91.6%) while yielding fewer invalid results (0.8%) than conventional swabs. By aligning with a familiar menstrual product, the DDT addresses usability concerns that hinder confidence in existing self-sampling devices, as evidenced by 70.5% participant preference in focus groups. These findings advance progress toward World Health Organisation (WHO) cervical cancer elimination targets by validating a culturally resonant, high-performance alternative to clinic-based sampling. The DDT’s potential to expand screening access, especially in low-resource settings or among individuals avoiding pelvic exams, could transform preventive care landscapes, reducing disparities in a disease rooted in healthcare inequity.

## INTRODUCTION

Cervical cancer is the fourth most common cancer among women worldwide, with an estimated 570,000 new cases and 311,000 deaths annually (1). This preventable cancer is caused by certain types of human papillomavirus (HPV). Cervical cancer screening programs are increasingly based on HPV testing, with a growing trend toward the use of self-sampling methods to identify high-risk HPV. HPV self-sampling can enhance participation rates and provides an opportunity to engage women beyond relying on traditional clinician-collected swabs (CCS) methods (2–8).

Studies have demonstrated that self-sampling is cost-effective compared to clinician sampling, with potential cost savings due to reduced need for clinical visits and staff time, supporting its implementation as a viable option in both high-income and low- and middle-income countries (2–4). A randomized study by Aarnio et al. found that self-sampling for HPV testing led to higher participation rates and more cases of high-grade cervical intraepithelial neoplasia (CIN2+) detected at a significantly lower cost compared to clinician-based sampling (5). Recent research in the United Kingdom (UK) found that 86% of women preferred having a choice between self-sampling and clinician sampling for cervical screenings, with 69% expressing a preference for self-sampling at home (8).

The WHO has set an ambitious goal to eliminate cervical cancer by the end of the century (9). However, this target faces significant challenges. In the UK, a high-income country that offers organized cervical screening, recent data show that only 68.7% of eligible individuals were adequately screened in 2021–2022, well below the 80% target (10). Additionally, in low- and middle-income countries where the majority of cancers manifest, screening programs are often absent. To achieve the goal of cervical cancer elimination, innovative approaches to screening are urgently needed. These must address both the barriers to participation and the resource limitations within healthcare systems. While vaginal self-sampling (VSS) has been shown to be an effective strategy for increasing cervical cancer screening participation, particularly among under-screened populations (9), some patients also report low self-confidence in obtaining a good quality vaginal sample with self-administered swabs, highlighting the need for more user-friendly self-sampling options (7).

Tampons, absorbent devices used primarily during menstruation, could offer a promising alternative for HPV sample collection due to their familiarity (10). Tampons may also have an advantage as sampling tools due to their ability to remain in the vaginal canal for an extended period and their larger size compared to other options (11, 12). While there are several studies that have assessed the analytical and clinical performance of VSS for HPV testing (6), limited research has been conducted on the performance of tampons as a self-collection device. A systematic review and meta-analysis by Arbyn et al. highlighted the need for further research to validate the use of tampons for HPV testing (6).

The Daye diagnostic tampon (DDT) is an innovative at-home gynecological test kit that utilizes a tampon for vaginal and cervical sample collection for high-risk HPV testing. Preliminary research has shown that self-collected tampons exhibit accuracy comparable to CCS for detecting HPV and other sexually transmitted infections (13). The present technical study builds on this preliminary data and was designed to compare the performance of the DDT to CCS for the detection of high-risk HPV; the influence of sample collection order on performance and associated comparison(s) was also assessed.

## MATERIALS AND METHODS

### Study design and setting

This study employed a prospective diagnostic trial design to assess the diagnostic accuracy of the DDT compared to CCS and self-collected VSS for the detection of high-risk HPV. As the performance of VSS and DDT may be affected by sampling order, block randomization was used to assign the order of self-sampling with DDT or VSS. Usability and acceptability were determined through questionnaires at baseline and after sampling. To further explore acceptability, participants were offered the opportunity to participate in focus groups after trial completion.

The trial was conducted in the UK between 19 December 2023 and 18 October 2024. Ethical approval was obtained from the London Camberwell St Giles Ethics Committee (reference 23-LO-0882). We report against the Standards for Reporting of Diagnostic Accuracy Studies guidelines (14). See Supplementary file 1 for the completed checklist.

### Study population

The study enrolled sexually active individuals assigned female at birth (AFAB) aged 25–65 years. Participants were eligible if they were sexually active, defined as having penetrative vaginal sex, and willing to provide informed consent and adhere to trial procedures. Individuals were ineligible if they had undergone a previous hysterectomy or total hysterectomy with cervix removal, had known allergies or sensitivities to tampons, or had a history of toxic shock syndrome. The study also excluded pregnant or breastfeeding individuals, those participating in other interventional clinical trials, and anyone using investigational drugs within the previous 30 days.

Eligible participants were divided into two groups, with group 1 consisting of individuals who had received a confirmed HPV-positive screening result within the past 4 weeks and could provide evidence through the trial electronic patient-reported outcome (ePRO) system. Group 2 did not have this additional eligibility criteria.

### Recruitment

**Fig 1:**
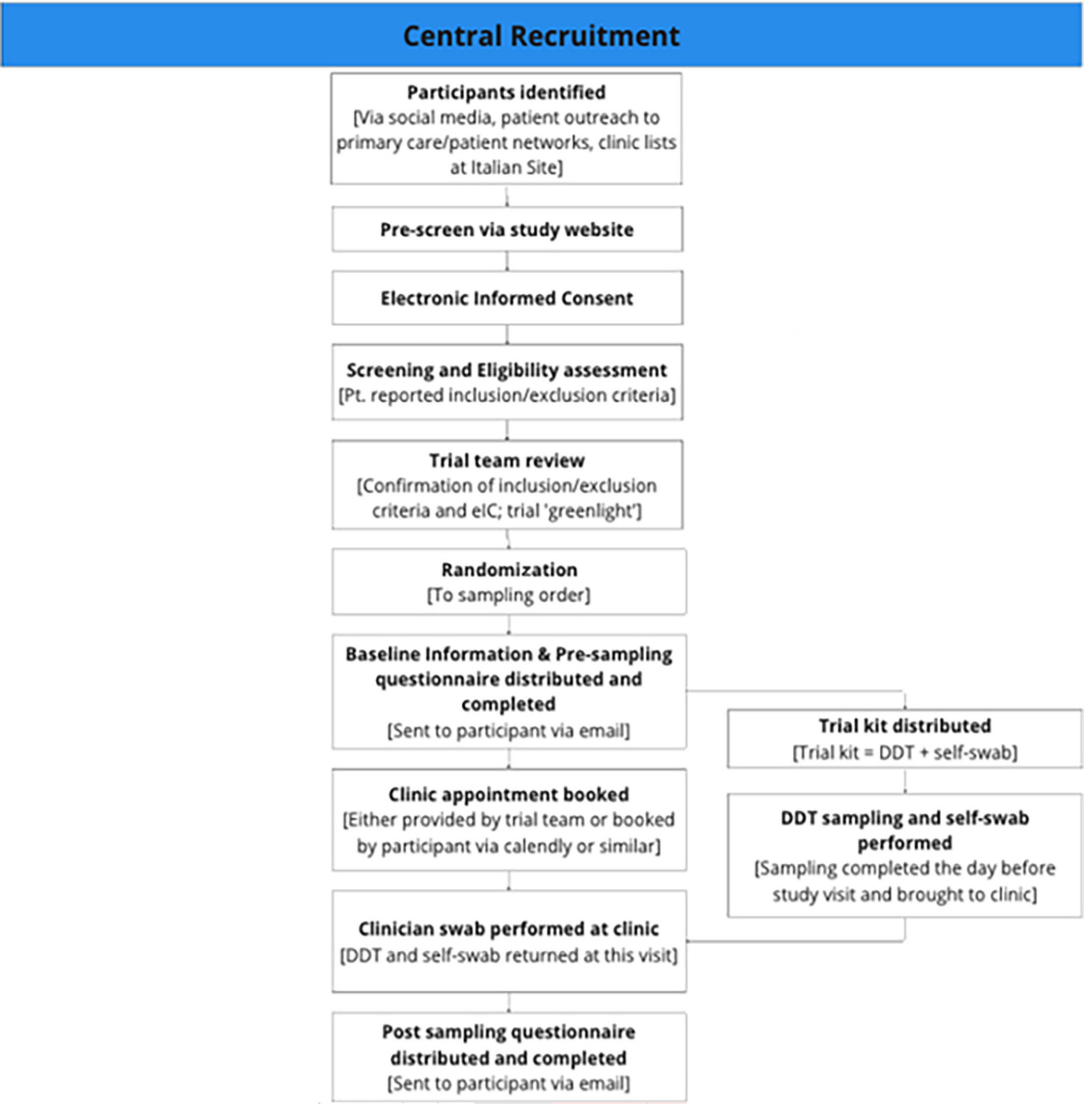
Central recruitment flow, illustrating the complete participant journey from initial identification through pre-screening, electronic consent, eligibility assessment, randomization, pre-sampling questionnaire completion, trial kit distribution, at-home sampling with the diagnostic tampon and self-swab, clinic appointment for clinician sampling, and post-sampling feedback.

Figure 1 depicts the central recruitment flow for the trial. Participants were primarily recruited via social media. Advertising on Meta and other platforms was used to highlight the trial. Between 15 January 2024 and 16 February 2024, adverts directed potential participants to the trial website (see Supplementary file 2). If interested in taking part, they completed an online pre-screening form to assess their eligibility for the trial. If potentially eligible, the participant information sheet and informed consent form were sent directly to the participant. Once enrolled, participants booked an in-person clinic appointment to see a trial nurse at a clinic. Participants self-sampled the day before their in-person clinic appointment. Participants were offered £50 to participate in the trial.

### Study procedures and sample collection

Participants underwent pre-screening, informed consent, screening, and eligibility assessments online, where baseline demographic information and medical history were collected. Participants were then randomly assigned to group A (self-swab followed by DDT) or group B (DDT followed by self-swab) using block randomization. Each participant received a trial kit by post, containing both the DDT and self-swab, along with sterile containers with transport medium and detailed sampling instructions.

Participants were instructed to use the DDT by inserting it into their vagina, leaving it in place for at least 20 minutes, then removing it before placement in a sterile container with 10 mL of transport medium (Copan UTM). The self-swab was also inserted vaginally and rotated before also being placed into a sterile container with 3 mL of transport medium (Copan UTM). The sampling order was specified in the instructions, and participants were asked to allow an hour between the first and second samples.

At a clinic visit the following day, participants provided their self-collected DDT and VSS samples to a nurse, and a clinician-collected vaginal swab was also obtained. After completing all samples, participants filled out additional questionnaires directly in an electronic data capture platform. Participants were offered the opportunity to participate in focus groups to discuss their experience of using DDT and their preferences for HPV testing. The trial period lasted approximately 2–4 weeks, with all samples sent to an accredited central UK laboratory for analysis.

### Sample collection devices

The vaginal swabs used for VSS and CCS were Copan FLOQSwabs, which consist of a customizable molded plastic shaft and a tip coated with perpendicular short Nylon fibers. Sterile containers for DDT and swab transportation contained 10 mL (DDT) and 3 mL (Swab) of transport medium (Copan UTM). Earlier optimization work using “spiked” tampons showed that 10 mL resuspension was viable and worked operationally when performing pre-analytical optimization. For resuspension of the swab, 3 mL was chosen on the basis of Connor et al. (15), which concluded that resuspension of the FLOQSwab in volumes ≤5 mL was optimal for downstream HPV testing (15).

**Fig 2:**
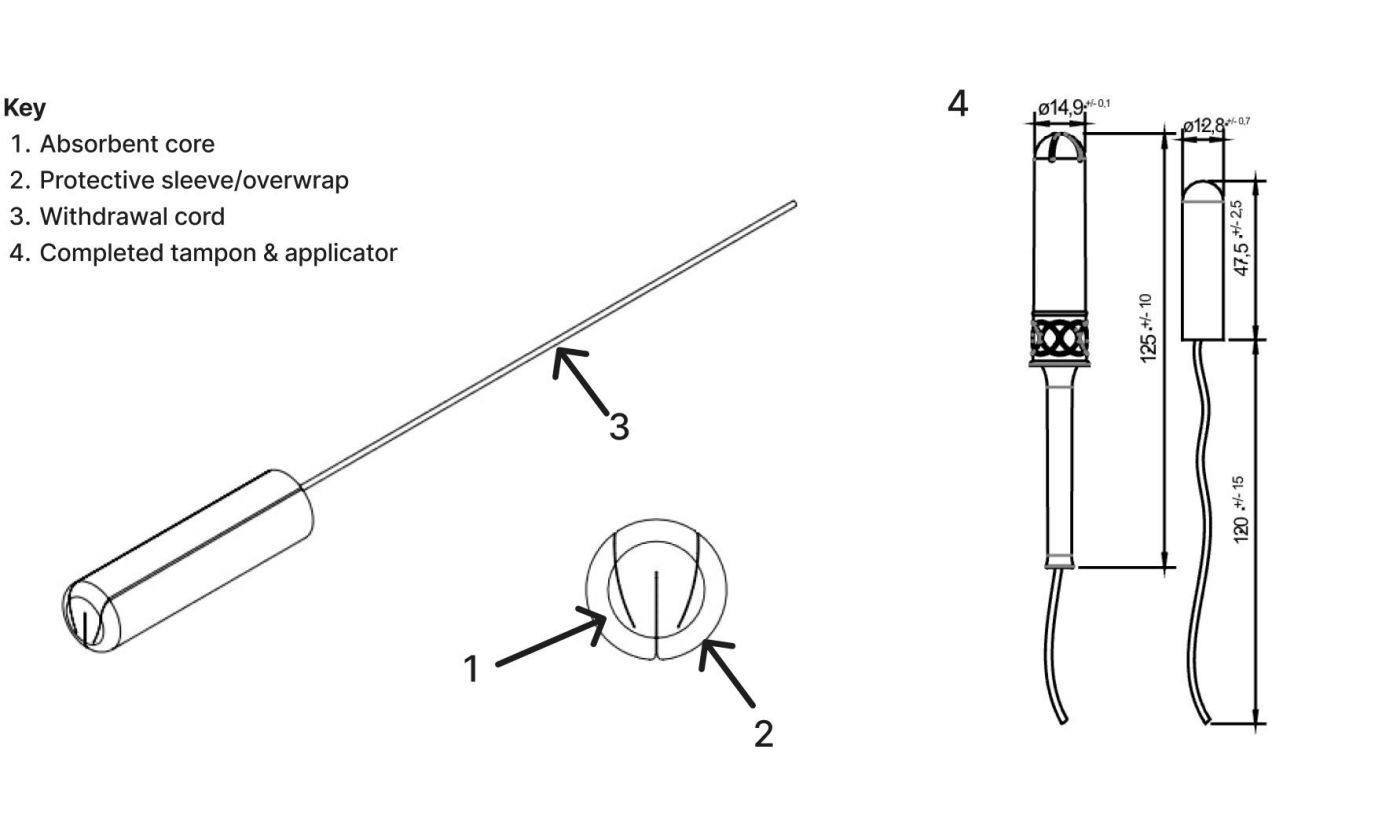
Structure, dimensions, and characteristics of the tampon as well as its raw materials

Diagnostic tampons used for self-sampling were sterile, 100% organic cotton, featuring a “W” wadding design with a protective sleeve and a bio-based polyethylene applicator (Fig. 2). The applicator ensures the patient can collect a sample from the cervical area, as well as from the vaginal canal. A withdrawal cord, made from mercerized organic cotton, is attached to the pledget. In the UK, the Daye diagnostic tampon is registered with the Medicines and Healthcare products Regulatory Agency (MHRA) and is Conformite Europeenne (CE) marked, and lab protocols are United Kingdom Accreditation Service (UKAS) and Care Quality Commission (CQC) accredited. In the United States, Daye is 510 k cleared, and lab protocols are Clinical Laboratory Improvement Amendments of 1988 (CLIA) and Certified Analytics Professional (CAP) certified. Furthermore, Daye is ISO13485 and Good Manufacturing Practices (GMP) certified and Food and Drug Administration (FDA) audited.

### HPV testing

The Aptima HPV assay (Hologic, Marlborough, MA, US) was utilized for HPV detection using the Panther platform. It is based on transcription-mediated amplification and detects the expression of E6/E7 mRNA of 14 high-risk HPV types (16, 18, 31, 33, 35, 39, 45, 51, 52, 56, 58, 59, 66, and 68). It is qualitative with detection levels calibrated to disease endpoints, and it has been shown to fulfill international acceptance criteria for use in cervical screening (16).

The HPV Aptima test has calibrators and internal control (IC) to monitor nucleic acid capture, amplification, and detection, as well as operator or instrument error. However, it does not have endogenous cellular housekeeping control. While the assay was performed according to the manufacturer’s instructions, the tampon and vaginal bio-samples used in the evaluation were not formally validated by the manufacturer.

Test results in the Panther are automatically determined by the assay software. The test results can be positive (for at least one of the types listed above), negative (for the types listed above), or invalid. An invalid result is reported if a sample tests negative for HPV and also negative for the IC, which acts as a “check” to ensure amplification has been successful. A low IC signal (below the cutoff), when combined with a negative result (S/CO < 0.50), may indicate sample degradation, inadequate specimen collection, inhibition, or poor storage conditions. While all are possible reasons for an invalid result, the most common reason is due to sample inhibition.

Following the Aptima instructions for use, invalid test results were repeated. If, following two invalid results of one sample type, the other two sample type(s) associated with a sampling episode provided two negative results, a negative result would be reported. Communication of positive results followed this same process; if two/three sample types provided positive results and one remained invalid after a repeat, a positive result would be reported. In instances where one sample type remained invalid on repeat, and the other two sample types were discrepant with the discrepancy unresolved through repeat testing, the results would be reported to the participant as inconclusive.

### Reference and index sampling methods

In this study, the gold standard or reference sampling method used was CCS, as used in many other self-sampling diagnostic accuracy studies. Our primary index sampling method of investigation was the DDT; however, we also calculated the accuracy of VSS as an alternate index. As an exploratory sensitivity analysis, we also explored a collated measure of results from CCS, VSS, and DDT as an alternative reference sampling method. With this collated measure, a positive result was recorded only when at least two sampling methods showed positive results. If only one of the three methods showed a positive result, the outcome was considered inconclusive. A negative result required all three samples to test negative.

### Statistical analyses

Diagnostic accuracy parameters (sensitivity, specificity, overall accuracy, positive, and negative predictive values) were calculated from all complete and conclusive results and reported with Wilson score 95% confidence intervals. Invalidity rates were compared across sampling methods.

Additional sensitivity analyses were conducted. To compensate for variation in conclusive results, diagnostic accuracy parameters were repeated using the collated measure as the gold standard. McNemar tests were conducted to assess whether diagnostic accuracy differed significantly between CCS and the collated measure as gold standards. To understand the impact of sampling order, a sensitivity analysis was conducted assessing diagnostic accuracy for tampon swabs and self-swabs when the respective sampling method was used first vs second. McNemar tests were conducted to compare diagnostic accuracy between DDT and VSS first orders for VSS and DDT, respectively.

### Sample size

A sample size of 420 participants was calculated to provide a statistical power of 90% to test whether the sensitivity of DDT is above 70% when the assumed true sensitivity is 95%, and the prevalence of HPV in the recruited patient population is 5%. This was assumed to yield 21 cases of HPV positivity to evaluate the sensitivity of HPV.

### Focus groups

Focus groups were held online via Google Meet, a video-calling platform. Participants were invited to participate via email following the completion of their clinic visit. Participants were offered an additional £25 to take part in these groups. Informed consent was taken before participation. The groups were facilitated by VM and MG, with support from another facilitator (MT). The topic guide can be found in supplementary material (Supplementary file 3).

## RESULTS

### Sample

**Fig 3:**
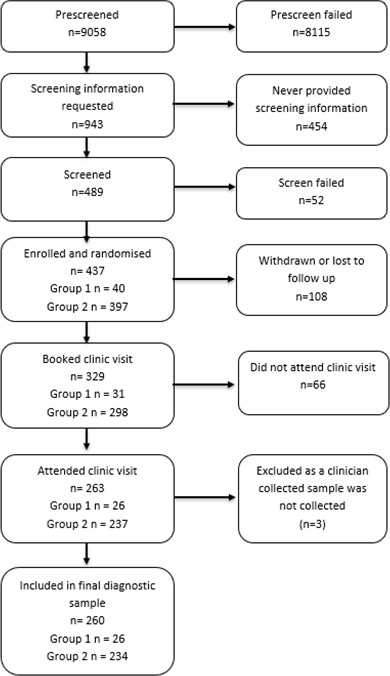
Participant flowchart.

From an initial screening of 489 participants, 437 were enrolled, with 52 screen failures. Of the 329 booked in for a clinic visit, 263 participants attended a clinic visit for CCS, having completed DDT and VSS and all questionnaires for acceptability outcomes. As three participants either did not complete CCS in a clinic or had missing CCS results, the final sample used for diagnostic accuracy results consisted of 260 participants who successfully completed all study requirements, with results from all three sampling methods (CCS, VSS, and DDT). In the final diagnostic accuracy sample, 26 participants had a confirmed HPV+ screening result within the previous 4 weeks (group 1). See Fig. 3 for the participant flow chart.

### Participant characteristics

The median age was 31.0 years, with an interquartile range between 28.0 and 36.0 years. Most participants were white (69%), heterosexual (75%), and single (59%, see Table 1). Characteristics of the sample used for diagnostic accuracy results (*n* = 260), as a whole and broken down by randomization arm, can be found in Supplementary file 4, Table 1.

**TABLE 1:** Participant characteristics

Characteristic	*N* = 263^*a*^
Age	31 (28, 36)
Ethnicity	
Asian	16 (6.1%)
Black, Caribbean, or African	25 (9.5%)
Other	39 (14.8%)
White	183 (69.6%)
Sexual orientation	
Heterosexual	197 (74.9%)
Bisexual	49 (18.6%)
Prefer not to say	7 (2.7%)
Lesbian/gay	6 (2.3%)
Other	4 (1.5%)
Relationship status	
Single	155 (58.9%)
Co-habiting	53 (20.2%)
Married	27 (10.3%)
Civil partnership	12 (4.6%)
Prefer not to say	10 (3.8%)
Divorced	6 (2.3%)

^*a*^ Median interquartile range(IQR); *n* (%).

### Diagnostic accuracy of DDT using CCS as the gold standard: primary outcome

A total of 780 samples (one sample per sampling method) from 260 participants were analyzed. As the primary outcome, diagnostic accuracy was determined using CCS as the gold standard.

For the CCS, 70 participants tested positive for HPV (26.7%), and 168 participants tested negative (64.6%). For 22 participants, the test result was invalid (8.5%). See Supplementary file 5, Tables 2 and 3.

**TABLE 2:** Diagnostic accuracy of DDT and VSS compared to CCS

	DDT^*a*^	VSS^*a*^
Sensitivity	82.9 (72.4–89.9)	75.4 (64.0–84.0)
Specificity	91.6 (86.4–94.9)	90.6 (85.1–94.2)
positive predictive values(PPV)	80.6 (70.0–88.0)	77.6 (66.3–85.9)
negative predictive value (NPV)	92.7 (87.7–95.8)	89.5 (83.8–93.3)
Accuracy	89.0 (84.4–92.4)	86.0 (80.9–89.9)

^*a*^ All values in percent. Values in parentheses indicate 95% CI limits.

**TABLE 3:** Diagnostic accuracy of CCS, DDT, and VSS compared to collated measure as reference

	CCS^*a*^	DDT^*a*^	VSS^*a*^
Sensitivity	95.4 (87.3–98.4)	92.5 (83.7–96.8)	84.6 (73.9–91.4)
Specificity	96.3 (92.2–98.3)	96.0 (91.9–98.1)	94.6 (90.1–97.1)
PPV	91.2 (82.1–95.9)	89.9 (80.5–95.0)	85.9 (75.4–92.4)
NPV	98.1 (94.6–99.4)	97.1 (93.4–98.8)	94.0 (89.4–96.7)
Accuracy	96.0 (92.6–97.9)	95.0 (91.5–97.1)	91.8 (87.6–94.7)

^*a*^ All values in percent. Values in parentheses indicate 95% CI limits.

Diagnostic accuracy metrics for both DDT and VSS vs CCS are reported in Table 2. Using the CCS as the gold standard yields a sensitivity of DDT of 82.9% (95% CI: 72.4%–89.9%), specificity of 91.6% (86.4%–94.9%), PPV of 80.6% (70.0%–88.0%), NPV of 92.7% (87.7%–95.8%), and overall accuracy of 89.0% (84.4%–92.4%). McNemar’s tests did not reveal a significant deviation of CCS and DTT results (*χ*^2^ = 0.04, df = 1, and *P* = 0.845) or of CCS and VSS results (*χ*^2^ = 0.03, df = 1, and *P* = 0.860), suggesting results do not differ in terms of sensitivity and specificity.

### Diagnostic accuracy of DDT using a collated measure as the gold standard: a sensitivity analysis

The proportion of conclusive results varied between the three sampling methods—CCS yielded results in 238 samples (90.8%), DDT in 258 (99.2%), and VSS in 248 (95.4%) out of 260 samples. Therefore, as an exploratory sensitivity analysis, we explored using a collated measure as the gold standard.

Using the collated measure as a reference, 67 participants were classified as positive for HPV (25.8%), 178 participants were categorized as negative (68.4%), and for two participants, the test result was invalid (0.8%), inconclusive (*n* = 14, 5.4%), or missing (*n* = 1, 0.4%).

CCS detected 62 of the 67 HPV-positive cases and 156 of the 178 HPV-negative samples (see Supplementary file 7, Table 4). Compared to the collated measure as a reference, CCS yielded a sensitivity of 95.4% (87.3%–98.4%), specificity of 96.3% (92.2%–98.3%), PPV of 91.2% (82.1%–95.9%), NPV of 98.1% (94.6%–99.4%), and overall accuracy of 96.0% (92.6%–97.9%; see Table 3). A McNemar’s test did not reveal a significant deviation of CCS and collated measure (*χ*^2^ = 0.44, df = 1, and *P* = 0.504).

**TABLE 4:** Diagnostic accuracy for VSS by sampling order

	VSS diagnostic accuracy when VSS is sampled first^*a*^	VSS diagnostic accuracy when DDT is sampled first^*a*^	*X* ^2^	*P*
Sensitivity	88.9 (74.7–95.6)	79.3 (61.6–90.2)	16.45	<0.001
Specificity	93.3 (85.3–97.1)	95.7 (89.3–98.3)	57.10	<0.001
PPV	86.5 (72.0–94.1)	85.2 (67.5–94.1)		
NPV	94.6 (86.9–97.9)	93.6 (86.8–97.0)		
Accuracy	91.9 (85.3–95.7)	91.7 (85.5–95.4)		

^*a*^ All values in percent. Values in parentheses indicate 95% CI limits.

Using DDT as a sampling method yielded correct positive results in 62 of 67 participants categorized as HPV-positive and correct negative results in 167 out of 176 HPV-negative participants, using the collated measure as a reference (see Supplementary file 8, Table 5). DDT sensitivity was 92.5% (95% CI: 83.7%–96.8%), specificity 96.0% (91.9%–98.1%), PPV 89.9% (80.5%–95.0%), NPV 97.1% (93.4%–98.8%), and overall accuracy 95.0% (91.5%–97.1%; see Table 3). A McNemar’s test did not reveal a significant deviation of DDT and the collated measure (*χ*^2^ = 0.08, df = 1, and *P* = 0.773). For VSS, diagnostic accuracy metrics are also reported in Table 3 (see Supplementary file 9, for an equivalent two-by-two table). A McNemar’s test did also not indicate a significant deviation of VSS and collated measure (*χ*^2^ < 0.01, df = 1, and *P* > 0.999).

**TABLE 5:** Diagnostic accuracy for tampon swabs by sampling order

	DDT diagnostic accuracy when DDT is sampled first^*a*^	DDT diagnostic accuracy when VSS is sampled first^*a*^	*X* ^2^	*P*
Sensitivity	100 (88.6–100)	86.5 (72.0–94.1)	30.03	<0.001
Specificity	96.8 (91.0–98.9)	95.0 (87.8–98.0)	65.62	<0.001
PPV	90.9 (76.4–96.9)	88.9 (74.7–95.6)		
NPV	100 (95.9–100)	93.8 (86.4–97.3)		
Accuracy	97.6 (93.1–99.2)	92.3 (86.0–95.9)		

^*a*^ All values in percent. Values in parentheses indicate 95% CI limits.

### Impact of sampling order on accuracy: a sensitivity analysis

#### Vaginal self-sampling

The sensitivity of VSS was higher when sampled first vs second (88.9% vs 79.3%, *χ*^2^ = 16.45, and *P* < 0.001), using the collated measure as the reference test (see Table 4). Specificity was slightly but significantly higher when DDT was sampled first compared to VSS first (self-swabs first: 93.3%, DDT first: 95.7%, *χ*^2^ = 57.10, and *P* < 0.001).

#### Daye diagnostic tampon

For DDT, sensitivity and specificity improved significantly when DDT was sampled first vs second (sensitivity: 100% vs 86.5%, *χ*^2^ = 30.03, and *P* < 0.001; specificity: 96.8% vs 95.0%, *χ*^2^ = 65.62, and *P* < 0.001; see Table 5).

### Acceptability

#### Questionnaire data

Participant questionnaire responses (*n* = 263) revealed high acceptance of tampon-based testing for HPV. Prior to sampling, 98.5% (259/263) had used tampons, but only 29.7% (78/263) were aware of their potential for diagnostic testing. Post-sampling tampons became the preferred testing method (46%), followed by self-swabs (33%). Comfort levels with tampon use for testing remained high (very comfortable: 206/263, 78.3% pre-sampling; 192/263, 73.0% post-sampling), and perceived ease of use increased from 63.5% (167/263) to 74.5% (196/263). While moderate or extreme concerns about accuracy slightly increased post-sampling (10.2% [27/263] to 13.6% [36/263], *P* < 0.05), overall acceptance remained strong. Trust in tampon results compared to clinician swabs remained stable.

#### Focus groups

Four focus groups were held with a total of 24 participants (5–7 participants in each group). Sessions lasted 60 minutes.

Of the 17 unique participants who stated preferences for HPV sampling methods during focus group discussions, 12 (70.5%) shared a strong preference for DDT. Four participants held more neutral positions, sharing mixed views and preferences between DDT, VSS, and visiting a clinic for sampling. One participant strongly preferred using clinic services. The participant who preferred clinic-based testing related this to their concerns about the accuracy and comfort of the DDT. Two other participants also had initial concerns about the accuracy of the DDT, despite their preference for it over other sampling methods. Other concerns raised in the group related to the convenience (1), cost (1), or sustainability (1) of the DDT or to the clarity of information provided around self-sampling with the DDT.

More extensive acceptability results from questionnaires and focus groups will be reported in a future publication.

## DISCUSSION

The primary objective of this study was to assess the accuracy of the DDT for detecting HPV infection. We found that, when using CCS as the reference standard, DDT exhibited a sensitivity of 82.9%, specificity of 91.6%, and overall accuracy of 89.0%. DDT diagnostic performance was as good as that of VSS, which had a sensitivity of 75.4%, a specificity of 90.6%, and an overall accuracy of 86.0% when compared with CCS. Results from questionnaires and focus groups suggest that tampons are an acceptable method of self-sampling for HPV, although comfort and perceived accuracy may be concerns for some.

The findings of this study align with the vast body of literature that exists about the comparable accuracy of VSS vs CCS (17, 18). A meta-analysis of six comparable studies to the present one, where VSS sought to identify the presence of HPV, rather than of precancerous cell changes (CIN2+), found a pooled sensitivity of 74% (61%–84%) and specificity of 88% (83%–92%) when compared to CCS (17). Limited published evidence exists on the diagnostic performance of tampons as self-sampling devices for high-risk HPV. Two relevant studies in South Africa assessed the acceptability and accuracy of tampon-based self-collection for high-risk HPV testing, compared to CCS, one study with women living with HIV (19), another with gynecology clinic attendees (20). Tampon self-sampling in the study of Adamson and colleagues (19) exhibited a sensitivity of 77% and specificity of 77% for detecting high-risk HPV (19), while the comparable sensitivity and specificity in Tiiti et al.’s study (2021) were 86% and 88%, respectively (20). Unlike another older study by McLarty et al. (21) which found tampons to have a high proportion (27%) of specimens of insufficient quality (21), the DDT only yielded 0.8% (2/260) invalid results when compared to CCS results. However, we accept that this discrepancy may be in part affected by the different criteria used to define invalidity across the studies. Speaking of invalid results across sampling methods in our study, all samples were stored in the same way and used the same UTM medium. Considering that FLOQSwabs, tampons, and UTM medium are not cleared for use with the Aptima HPV test, we hypothesize that variation in inhibition could be due to sample collection devices.

Sensitivity analyses showed that the diagnostic performance of the DDT increased to a sensitivity of 92.5% (83.7%–96.8%), a specificity of 96.0% (91.9%–98.1%), and an overall accuracy of 95.0% (91.5%–97.1%) when using the collated measure as the reference standard, rather than CCS. We conducted the sensitivity analysis with a collated measure to mitigate against solely relying on the CCS given that it produced the highest invalidity rate. We suspect that the robustness of CCS in our study is limited by the fact that the swab used for both CCS and VSS is not formally approved for use for APTIMA testing. As the APTIMA HPV test used does not include an endogenous cellular control, invalidity was driven by other reasons beyond a lack of available cellular material, which likely included inhibition. The novel approach of using a composite measure leverages all available information and mitigates against the high invalidity rate from CCS alone. Notably, DDT and CCS performed very similarly when using the collated measure.

Sensitivity analyses also suggest that sampling order may have had an impact on DDT’s diagnostic performance. The analysis of sampling order revealed significant effects of the order of sampling on diagnostic accuracy for VSS and DDT. Sensitivity increased for both methods when sampled first, which is consistent with expectation and published evidence. Specificity decreased slightly (but significantly) for VSS when the VSS was sampled first; interestingly, specificity was increased when the DDT was taken first. Reasons for the specificity observations are not entirely clear; while the dimensions and absorbency of the different devices are likely to play a role, these data underline the importance of the evaluation and validation of individual devices.

Finally, we found the DDT to be an acceptable method for HPV self-sampling. This echoes the few other UK-based and global studies conducted, which have found it to be an acceptable, convenient, and easy-to-use option (19, 20, 22). A study in London (*n* = 501) where women were recruited from colposcopy units found the majority of participants (98%) approved of tampons being used as a self-collection tool to test for HPV infection (22). The above-mentioned South African studies also reported high levels of acceptability. Adamson et al.’s study (2015) of women living with HIV (*n* = 325) found that over 90% of participants reported no difficulties in collecting HPV samples with a tampon, and 82% were willing to self-collect with a tampon at home (19). Another study in South Africa (*n* = 527) found very similar results showing self-sampling with a tampon to be a comfortable, painless option, preferable for many (20).

### Study strengths and limitations

This is a timely study of an innovative device that could broaden choice within HPV self-sampling (9, 10). It is important to note the contribution this study makes to a gap in evidence, as there are limited data on the acceptability and performance of tampons as biospecimens for HPV testing, in particular where the impact of sampling order is assessed (19, 20, 22). Consequently, we hope these data act as a grounding for further development and evaluation of the DDT.

Some limitations should be considered. We experienced a greater loss to follow-up than anticipated, resulting in a smaller sample than planned of 260. However, due to targeted recruitment of those with a confirmed HPV result, which enriched our population, we obtained the required number of cases to evaluate the DDT sensitivity if greater than 70%, compared to CCS. In this study, we only used one HPV assay; further work could enhance findings by evaluating other DDT and HPV assay combinations, increasing reliability. We recognize that the Aptima HPV assay is not formally validated for tampon or vaginal samples. However, there remains a relatively limited number of assays formally validated for self-taken samples compared to clinician-taken samples (23), and several studies suggest biological equivalence of HPV detection in vaginal vs cervical samples (24, 25). Certainly, future research should address this validation gap and define further parameters to support pre-analytical processing of tampons. Additional research comparing the quality and yield of biomaterial collected across sampling devices at the qualitative level and the type-specific level would also be of value, and work is in train to address this. As mentioned, despite being the commonly referenced gold standard, CCS produced the highest invalidity rate among the three sampling methods. To mitigate against this, we conducted a sensitivity analysis with a collated measure. Most importantly, we recognize that our assessment focused on diagnostic accuracy for HPV detection, rather than HPV associated with histologically confirmed disease, specifically CIN2+. The absence of long term follow-up limits our ability to assess the sampling methods’ longitudinal performance (26). To address this, ongoing work includes the evaluation of the clinical performance of the DDT relative to CIN2+, which will naturally extend the data presented here. Additionally, it is crucial to assess the effectiveness of DDT in diverse populations, considering various age groups, ethnicities, and socioeconomic backgrounds, to ensure broad applicability and address potential confounding factors (27). While we did achieve a diverse sample in terms of ethnicity and sexuality, which is of particular importance due to lower screening in these groups (28, 29), future studies could be repeated with older participants. This is particularly important given that cervical cancer screening programs in the UK suggest screening until women and individuals AFAB reach 64 years old (30).

### Conclusion

This study demonstrates the credibility of using diagnostic tampons as an alternate method to CCS for detecting high-risk HPV infections. Notably, DDT was performed similarly to CCS when using the collated measure as the reference standard. The performance and ease of use of DDT highlight its potential as a self-collection method for cervical cancer screening. Further research is needed to validate these findings in longitudinal cohorts and relative to clinical endpoints. Understanding the practical challenges and logistical considerations of DDT implementation, as well as comprehensive cost-effectiveness analyses, will help determine the financial viability of integrating DDT into screening programs, particularly in low-income countries (31).

## Data Availability

Both the full study protocol and the study data are available on reasonable request, with requests made to the corresponding author.

## SUPPLEMENTAL MATERIAL

Supplemental materials jcm.01852-24-s0001.docx Supplemental materials and Tables S1 to S7.

ASM does not own the copyrights to Supplemental Material that may be linked to, or accessed through, an article. The authors have granted ASM a non-exclusive, world-wide license to publish the Supplemental Material files. Please contact the corresponding author directly for reuse.
