# Supplementary material for: Diagnostic accuracy of the Daye diagnostic tampon compared to clinician-collected and self-collected vaginal swabs for detecting HPV: a comparative study
Source: J Clin Microbiol. 2025 Apr 11;63(5):e01852-24. doi: 10.1128/jcm.01852-24 (PMC12077143; doi:10.1128/jcm.01852-24)
Supplement: Supplemental materials — and Tables S1 to S7. [file jcm.01852-24-s0001.docx]

**Supplementary material**

**Supplementary material 1. STARD checklist**

| Section and Topic | No. | Item | Location in manuscript |
| --- | --- | --- | --- |
| Title or abstract |  |  |  |
|  | 1 | Identification as a study of diagnostic accuracy using at least one measure of accuracy (such as sensitivity, specificity, predictive values, or AUC) | Page 1 |
| Abstract |  |  |  |
|  | 2 | Structured summary of study design, methods, results, and conclusions (for specific guidance, see STARD for Abstracts) | Page 1-2 |
| Introduction |  |  |  |
|  | 3 | Scientific and clinical background, including the intended use and clinical role of the index test | Page 2-3 |
|  | 4 | Study objectives and hypotheses | Page 3 |
| Methods |  |  |  |
| Study design | 5 | Whether data collection was planned before the index test and reference standard were performed (prospective study) or after (retrospective study) | Page 3 |
| Participants | 6 | Eligibility criteria | Page 3-4 |
|  | 7 | On what basis potentially eligible participants were identified (such as symptoms, results from previous tests, inclusion in registry) | Page 3 |
|  | 8 | Where and when potentially eligible participants were identified (setting, location and dates) | Page 4 |
|  | 9 | Whether participants formed a consecutive, random or convenience series | Page 4 |
| Test methods | 10a | Index test, in sufficient detail to allow replication | Page 5-6 |
|  | 10b | Reference standard, in sufficient detail to allow replication | Page 5-6 |
|  | 11 | Rationale for choosing the reference standard (if alternatives exist) | Page 6 |
|  | 12a | Definition of and rationale for test positivity cut-offs or result categories of the index test, distinguishing pre-specified from exploratory | Page 6 |
|  | 12b | Definition of and rationale for test positivity cut-offs or result categories of the reference standard, distinguishing pre-specified from exploratory | Page 6 |
|  | 13a | Whether clinical information and reference standard results were available to the performers/readers of the index test | Page 5 |
|  | 13b | Whether clinical information and index test results were available to the assessors of the reference standard | Page 5 |
| Analysis | 14 | Methods for estimating or comparing measures of diagnostic accuracy | Page 6-7 |
|  | 15 | How indeterminate index test or reference standard results were handled | Page 6 |
|  | 16 | How missing data on the index test and reference standard were handled | Page 7 |
|  | 17 | Any analyses of variability in diagnostic accuracy, distinguishing pre-specified from exploratory | Page 9-10 |
|  | 18 | Intended sample size and how it was determined | Page 7 |
| Results |  |  |  |
| Participants | 19 | Flow of participants, using a diagram | Figure 3, page 8 |
|  | 20 | Baseline demographic and clinical characteristics of participants | Table 1, page 8 |
|  | 21a | Distribution of severity of disease in those with the target condition | N/A |
|  | 21b | Distribution of alternative diagnoses in those without the target condition | Page 0 |
|  | 22 | Time interval and any clinical interventions between index test and reference standard | Page 5 |
| Test results | 23 | Cross tabulation of the index test results (or their distribution) by the results of the reference standard | Supplementary material |
|  | 24 | Estimates of diagnostic accuracy and their precision (such as 95% confidence intervals) | Table 3, page 10; page 12 |
|  | 25 | Any adverse events from performing the index test or the reference standard | Page 9 |
| Discussion |  |  |  |
|  | 26 | Study limitations, including sources of potential bias, statistical uncertainty, and generalisability | Page 15 |
|  | 27 | Implications for practice, including the intended use and clinical role of the index test | Page 15 |
| Other information |  |  |  |
|  | 28 | Registration number and name of registry | Page 17 |
|  | 29 | Where the full study protocol can be accessed | Page 17 |
|  | 30 | Sources of funding and other support; role of funders | Page 17 |

Bossuyt, P.M., Reitsma, J.B., Bruns, D.E., Gatsonis, C.A., Glasziou, P.P., Irwig, L., et al. (2015). STARD 2015: An updated list of essential items for reporting diagnostic accuracy studies

**Supplementary material 2. Social media recruitment adverts**


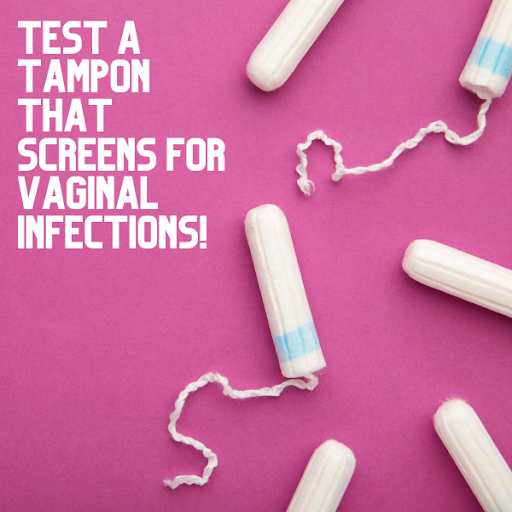

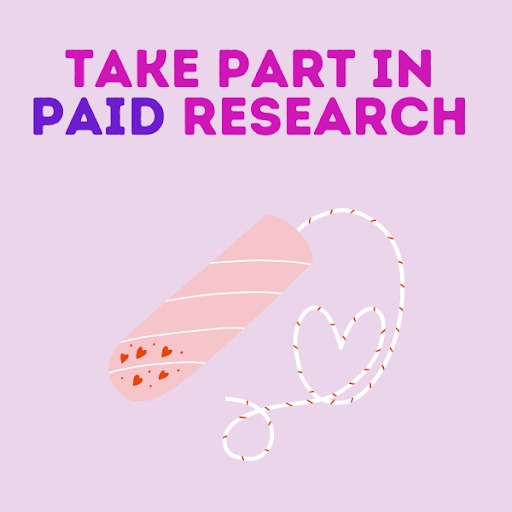


**Supplementary material 3. Focus group topic guide**

1. Motivation for Joining:

- What motivated you to participate in the STAMP trial?
- What is your previous experience with HPV testing?
- What was your experience with the communication and logistics surrounding the trial?

1. Experience with Collection Methods:

- Can you describe your experience with the self-collected tampon? Compared to the other
- two methods
- are you familiar with tampons already? Applicator vs non applicator?
- What challenges did you face during the sample collection process?

1. Usability of the DT:

- How did you find the instructions for using the tampon?
- do you have experience with at home testing kits?
- what are your perceptions of at home testing kits?
- did you have any concerns about doing the self sampling yourself?
- What improvements would you suggest for the sample collection process with the DT?

1. Comfort Using the DT:

- How comfortable were you using the tampon for sample collection?
- What could be done to improve your comfort level?
- Did you feel anxious prior to sample collection?

1. Acceptability and Perceptions:

- How confident do you feel about using a tampon for sample collection in terms of
- accuracy?
- If the tampon was offered by the NHS, would you be more confident about it?
- Which sample collection method would you prefer for future tests? Factors that influence
- Would you recommend the tampon-based sampling to others? Why or why not?

**Supplementary material 4. Table 1. Participant characteristics of diagnostic accuracy analyses (n=260), as a whole and by randomisation arm**

| **Characteristic** | **DDT first, N = 132**^1^ | **VSS first, N = 128^1^** | **Overall, N = 260^1^** |
| --- | --- | --- | --- |
| Age | 32 (28, 37) | 31 (28, 36) | 31 (28, 36) |
| **Ethnicity** |  |  |  |
| White | 93 (70%) | 87 (68%) | 180 (69%) |
| Other | 19 (14%) | 20 (16%) | 39 (15%) |
| Black, Caribbean or African | 14 (11%) | 11 (8.6%) | 25 (9.6%) |
| Asian | 6 (4.5%) | 10 (7.8%) | 16 (6.2%) |
| **Sexual Orientation** |  |  |  |
| Heterosexual | 99 (75%) | 96 (75%) | 195 (75%) |
| Bisexual | 27 (20%) | 21 (16%) | 48 (18%) |
| Prefer not to say | 2 (1.5%) | 5 (3.9%) | 7 (2.7%) |
| Lesbian/Gay | 2 (1.5%) | 4 (3.1%) | 6 (2.3%) |
| Other | 2 (1.5%) | 2 (1.6%) | 4 (1.5%) |
| **Relationship Status** |  |  |  |
| Single | 78 (59%) | 75 (59%) | 153 (59%) |
| Co-habiting | 26 (20%) | 26 (20%) | 52 (20%) |
| Married | 12 (9.1%) | 15 (12%) | 27 (10%) |
| Civil Partnership | 6 (4.5%) | 6 (4.7%) | 12 (4.6%) |
| Prefer not to say | 6 (4.5%) | 4 (3.1%) | 10 (3.8%) |
| Divorced | 4 (3.0%) | 2 (1.6%) | 6 (2.3%) |
| HPV Infection in last 6 Months | 29 (22%) | 34 (27%) | 63 (24%) |

1 Median (IQR); n (%)

**Supplementary material 5. Table 2: DDT vs. CCS results**

|  |  | Daye Diagnostic Tampon (Index Result) | | | |
| --- | --- | --- | --- | --- | --- |
|  |  | Positive | Negative | Invalid | Total |
| Clinician collected sampling (Reference Result) | Positive | 58 | 12 | 0 | 70 |
|  | Negative | 14 | 153 | 1 | 168 |
|  | Invalid | 3 | 18 | 1 | 22 |
|  | Total | 75 | 185 | 2 | 260 |

**Supplementary material 6. Table 3: VSS vs. CCS results**

|  |  | Vaginal Self-Swab (Index Result) | | | |
| --- | --- | --- | --- | --- | --- |
|  |  | Positive | Negative | Invalid | Total |
| Clinician collected sampling (Reference Result) | Positive | 52 | 17 | 1 | 70 |
|  | Negative | 15 | 145 | 8 | 168 |
|  | Invalid | 3 | 14 | 5 | 22 |
|  | Total | 76 | 178 | 14 | 260 |

**Supplementary material 7. Table 4: CCS vs. collated measure**

|  |  | Clinician-collected swab (Index Result) | | | |
| --- | --- | --- | --- | --- | --- |
|  |  | Positive | Negative | Invalid | Total |
| Collated measure (Reference Result) | Positive | 62 | 3 | 2 | 67 |
|  | Negative | 6 | 156 | 14 | 176 |
|  | Inconclusive | 2 | 8 | 4 | 14 |
|  | Invalid | 0 | 0 | 2 | 2 |
|  | Missing | 0 | 1 | 0 | 1 |
|  | Total | 70 | 168 | 22 | 260 |

**Supplementary material 8. Table 5: DDT vs. collated measure**

|  |  | Daye Diagnostic Tampon (Index Result) | | | |
| --- | --- | --- | --- | --- | --- |
|  |  | Positive | Negative | Invalid | Total |
| Collated measure (Reference Result) | Positive | 62 | 5 | 0 | 67 |
|  | Negative | 7 | 167 | 2 | 176 |
|  | Inconclusive | 6 | 8 | 0 | 14 |
|  | Invalid | 0 | 2 | 0 | 2 |
|  | Missing | 0 | 1 | 0 | 1 |
|  | Total | 75 | 183 | 2 | 260 |

**Supplementary material 9. Table 6: VSS vs. collated measure**

|  |  | Vaginal Self-Swab (Index Result) | | | |
| --- | --- | --- | --- | --- | --- |
|  |  | Positive | Negative | Invalid | Total |
| Collated measure (Reference Result) | Positive | 55 | 10 | 2 | 67 |
|  | Negative | 9 | 158 | 9 | 176 |
|  | Invalid | 0 | 0 | 2 | 2 |
|  | Inconclusive | 5 | 8 | 1 | 14 |
|  | Missing | 1 | 0 | 0 | 1 |
|  | Total | 70 | 176 | 14 | 260 |

**Supplementary material 10. Table 7: Acceptability results**

|  | Pre-sampling questionnaire (n=263), n (%) | Post-sampling questionnaire (n=263), n (%) |
| --- | --- | --- |
| Tampon use prior to trial | 259 (98.5) |  |
| Aware tampons could be used for testing for HPV, STIs, or BV | 78 (29.7) |  |
| Level of comfort with idea of using a tampon for sample collection |  |  |
| Very comfortable | 206 (78.3) | 192 (73.0) |
| Comfortable | 51 (19.4) | 49 (18.6) |
| Neither comfortable nor uncomfortable | 6 (2.3) | 5 (1.9) |
| Uncomfortable |  | 5 (1.9) |
| Very uncomfortable |  | 12 (4.5) |
| Perceived ease of tampon use for HPV sample collection |  |  |
| Very Easy | 167 (63.5) | 196 (74.5) |
| Easy | 84 (31.9) | 54 (20.5) |
| Neither easy nor difficult | 7 (2.7) | 8 (3.0) |
| Difficult |  | 3 (1.1) |
| Very difficult | 5 (1.9) | 2 (0.7) |
| Concerns about accuracy |  |  |
| Not at all concerned | 69 (26.2) | 63 (23.9) |
| Slightly concerned | 98 (37.3) | 115 (43.7) |
| Somewhat concerned | 69 (26.2) | 49 (18.6) |
| Moderately concerned | 19 (7.2) | 25 (9.5) |
| Extremely concerned | 8 (3.0) | 11 (4.1) |
| Trust in tampon results compared to clinician swabs |  |  |
| Equal trust in tampon and clinician swabs | 151 (57.4) | 144 (54.7) |
| Greater trust in clinician swab | 106 (40.3) | 114 (43.3) |
| Greater trust in tampon | 6 (2.3) | 5 (1.9) |
